# Characterization of Humoral Responses Induced by an H7N9 Influenza Virus-Like Particle Vaccine in BALB/C Mice

**DOI:** 10.3390/v7082821

**Published:** 2015-08-04

**Authors:** Li Zhang, Jing Lu, Yin Chen, Fengjuan Shi, Huiyan Yu, Chao Huang, Lunbiao Cui, Zhiyang Shi, Yongjun Jiao, Yuemei Hu

**Affiliations:** 1 Department of Vaccine Clinical Evaluation, Jiangsu Provincial Center for Disease Prevention and Control, Nanjing 210009, China; E-Mail: zhangli411@yeah.net; 2 Department of HIV/STD prevention and control, Jiangsu Provincial Center for Disease Prevention and Control, Nanjing 210009, China; E-Mail: hhtt319@163.com; 3 Institute of Pathogenic Microbiology, Jiangsu Provincial Center for Disease Prevention and Control, Key Laboratory of Enteric Pathogenic Microbiology, Ministry Health, Nanjing 210009, China; E-Mails: chenyin1231@foxmail.com (Y.C.); shifengjuan905@163.com (F.S.); yuhy86@126.com (H.Y.); nestar1987@126.com (C.H.); lbcui@njmu.edu.cn (L.C.); shizhiyang@jscdc.cn (Z.S.)

**Keywords:** H7N9 avian influenza, virus-like particles, vaccine, cross-reactivity, neutralizing antibodies

## Abstract

In April 2013, human infections with a novel avian influenza (H7N9) virus emerged in China. It has caused serious concerns for public health throughout the world. However, there is presently no effective treatment, and an A (H7N9) H7 subtype influenza vaccine is not available. Vaccination with virus-like particles (VLPs) has showed considerable promise for many other subtype influenza viruses. To produce H7N9 VLPs, full length, unmodified hemagglutinin (HA), neuraminidase (NA), and matrix1 (M1) genes from the A/Wuxi/1/2013(H7N9) were cloned into a pCDNA5.1 FRT vector. By co-transfection, VLPs containing HA, NA, and M1 were secreted by 293T cells. VLPs were purified by ultracentrifugation and injected into mice by the intramuscular route. In animal experiments, humoral and cellular immunoresponse were all triggered by H7N9 VLPs. High levels of specific antibodies and the isotypes of IgG were detected by ELISA. Anamnestic cellular immune responses were examined by detecting specific cytotoxic T cell for IFN-γ production in ELISPOT assay. The hemagglutination-inhibition (HAI) against the homologous virus was more than 1:64, and cross-reactive HAI titers against the heterologous virus (H1N1 and H3N2) were more than 1:16. Moreover, VLPs immunized mice showed a rapid increase of neutralizing antibodies, with neutralizing antibody titers more than 1:8, which increased four-fold against PBS immunized mice in week four. By week six, the mice had high neutralization ability against the given strain and held a potent homologous virus neutralizing capacity. Thus, VLPs represent a potential strategy for the development of a safe and effective vaccine against novel avian influenza (H7N9) virus.

## 1. Introduction

In April 2013, human infections with a novel avian influenza (H7N9) virus were reported in China. It has caused serious concerns for public health throughout the world [1,2,3]. The World Health Organization (WHO) has identified H7N9 as an “unusually dangerous virus for humans”. Most of the cases resulted in severe respiratory illness, and have a mortality rate of roughly 30 percent in hospitalized patients [4,5]. Researchers have provided comments on the unusual prevalence of older males among H7N9-infected patients. To date, the reason is unknown [6].

As for other types of influenza, vaccination is considered to be the most effective measure to prevent or mitigate a pandemic. As with other subtype influenza viruses, vaccination is considered to be the most effective measure to control the pandemic. However, some experts believe that there would be great difficulty in providing adequate supplies of a vaccine if the virus were to develop into a pandemic. Even with extensive antigenic drift and vaccine manufacturing capacity, the global public health community is concerned with the effectiveness of the traditional vaccines, particularly in persons older than 65 years [7]. The problems also address the high pathogenicity of H7N9 influenza virus [8], the need for biosafety-level 3 (BSL-3) containment facilities, and the low immunogenicity of H7N9 virions [9].

In order to address these obstacles, new strategies for rapid production of H7N9 influenza vaccines are a priority for pandemic preparedness. Influenza VLPs are produced by a self-assembly process when matrix protein 1 (M1), hemagglutinin (HA) and neuraminidase (NA) proteins are co-expressed [10]. VLPs, which are similar to infectious virions in the morphological and structural features, are non-infectious particles and have advantages in safety and manufacturing [11]. Influenza VLPs have been generated from various strains of virus including H1N1 [12], H3N2 [13], H5N1 [14,15,16], H9N2 [17], and H7N9 [18,19]. Recombinant VLPs can be efficiently absorbed, internalized and processed by antigen presenting cells (APCs), and capable of eliciting strong immune responses against viruses [20,21,22,23].

VLPs can be produced in multiple expression systems such as *E. coli* [24], yeast [25], baculovirus [11,12,16,18], and mammalian cells [26]. Most research about influenza VLP has focused on the baculovirus *Spodoptera frugiperda (Sf9)* expression system. In this report, we describe the development of an H7N9 influenza VLP comprised of HA, NA and M1 derived from avian influenza A/Wuxi/1/2013 (H7N9), by using mammalian cells. The H7N9 VLPs derived from 293T cells elicited hemagglutination-inhibition, neutralization activities, and cross-reactive in BALB/c mice. These results indicate that VLPs represent a promising vaccine candidate for H7N9 influenza and other subtypes of avian influenza viruses with pandemic potential.

## 2. Results

### 2.1. Production and Characterization of VLPs

**Figure 1:**
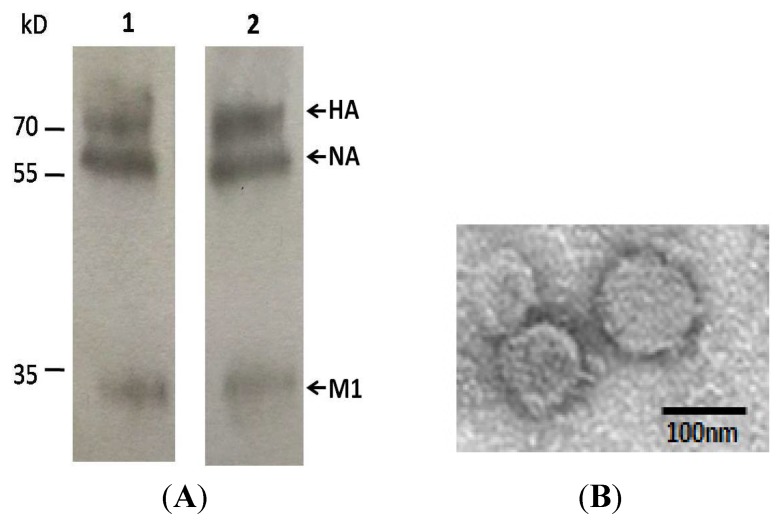
Generation of H7N9 avian influenza virus-like particles. (**A**) Analysis of virus-like particles (VLPs) in culture supernatants by Western blotting using H7N9 infected patient serum (lane 1) and mouse serum immunized by H7N9 virions (lane 2) to identify the expression of particles. Three expected bands with the molecular weight of 75 kD, 68 kD, and 28 kD, are equal to the size of hemagglutinin (HA), neuraminidase (NA), and matrix 1 (M1) of VLPs; (**B**) Identification of H7N9 avian influenza VLPs by transmission electron microscopy. Transfected 293T cells were fixed, dehydrated, embedded, stained with lead citrate and uranyl acetate, and observed by electron microscopy.

To generate H7N9 influenza VLPs, three recombinant plasmids encoding HA, NA, and M1 full-length genes were constructed respectively, and co-transfected into 293T cells. To identify the VLPs’ secretion ability of transiently transfected cells, culture supernatants were used to run SDS-PAGE, and transferred to nitrocellulose membrane. Membranes were incubated with H7N9-immunized mice sera and infected human sera, respectively, in Western blot analysis. As shown in Figure 1A, three bands with sizes of 75 kD, 68 kD, and 28 kD were confirmed by Western blot using H7N9 infected patients serum and mouse serum immunized by inactivated H7N9 virus. It demonstrated that HA, NA, and M1 of VLPs were successfully expressed as expected.

To further confirm the formation of self-assembled VLPs, the supernatant of transfected 293T cells was characterized by electron microscopy. As shown in Figure 1B, the morphology of VLPs resembles the morphology of influenza virus particles with spikes on their surfaces, characteristic of influenza virus HA proteins on virions. Particle sizes ranged from approximately 100 to 120 nm. After purification by ultracentrifugation, VLPs were collected. Total protein concentrations were determined by Pierce BCA Protein Assay Kit (Thermo, cat.: 23225) and the purity of VLPs was estimated by SDS-PAGE to be about 80%.

Taken together, we successfully acquired H7N9 influenza virus VLPs, which consisted of major antigenic proteins of the virus and exhibited similar morphological features as natural virus particles.

### 2.2. Antibody Responses Induced by Immunizations

**Figure 2:**
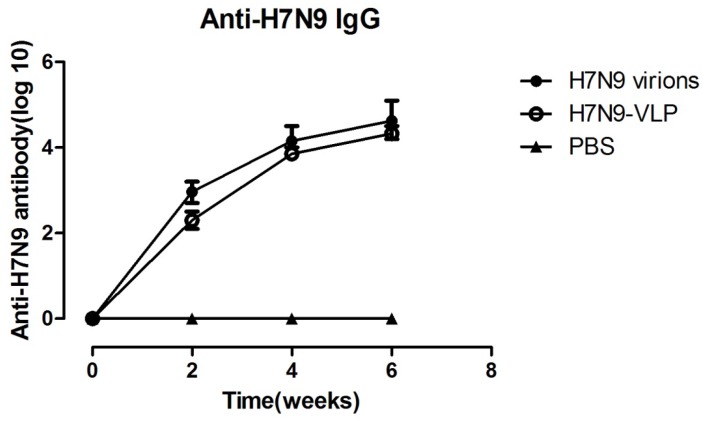
Virus specific IgG were enhanced by H7N9 VLPs or virions. BALB/c mice were vaccinated via intramuscular injection at weeks 0, 2, and 4 with 40 μg of influenza VLPs and WIV or PBS (negative control) only. 96-well plates were coated with inactivated virus, and serum of each group (n = 8) was tested for anti-H7N9 virions specific IgG by ELISA. Data was expressed as mean titer with a standard deviation (SD) bar. H7N9 virions, VLP and PBS: H7N9 virions, H7N9 VLPs, and PBS vaccinated mice serum.

To evaluate humoral responses induced by recombinant VLPs, BALB/c mice were immunized with 40 μg of VLPs three times, at two-week intervals. As shown in Figure 2, in comparison to the control, which immunized with PBS, VLPs elicited significant increase in antibody titer with immunization in mice. At week 6, the average antibody endpoint dilution titer (>1:60,000), and the magnitude of humoral immune responses induced by VLPs was similar to those induced by whole influenza virions (WIV).

**Figure 3:**
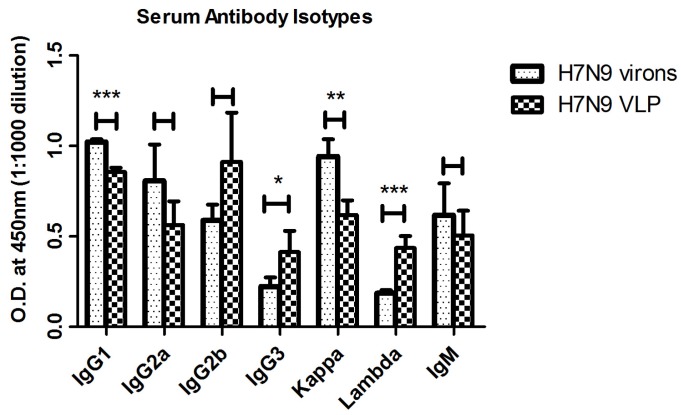
The antibody isotype of the anti-H7N9 virus antisera. Mice serum was collected from H7N9 virions, H7N9 VLP, and PBS immunized groups (n = 8) at week 6 post vaccination. Inactivated virus was coated in 96-well plates, and the heavy chain (including IgG1, IgG2a, IgG2b, IgG3, and IgM) and light chain (including kappa and lambda) isotypes in antisera were tested by ELISA at a dilution of 1:1000 and reported as specific O.D. H7N9 virions, VLP and PBS: H7N9 virions, H7N9 VLPs, and PBS vaccinated mice serum. *, **, and *** indicate statistical significance (*p* < 0.05, *p* < 0.01, and *p* < 0.001, respectively).

To further characterize the kinetics of antibody production, titers of serum antibody isotypes were determined at week 6. The dominant serum IgG heavy chain isotype subclasses elicited by VLPs were IgG2b, IgG1 and IgG2a, occupying 33.2%, 31.1% and 20.5%, respectively, of total compounds. In contrast, the predominant isotypes of mice vaccinated with WIV were IgG1 (38.7%), Ig2a (30.6%) and IgG2b (22.2%). IgG2a and IgG2b were associated with a dominant Th1 immune response, while IgG1 was indicative of a Th2 response. By statistical analysis, there was no statistically significant difference between VLPs and WIV in the antibody heavy chain isotypes. It indicated that no significant difference was observed between VLPs and WIV in cellular and humoral immunoresponse. However, with respect to the isotype of light chains, significant differences were detected in both kappa (*p* < 0.01) and lambda (*p* < 0.001) between VLPs and WIV (Figure 3).

### 2.3. H7N9 VLPs Induced Virus-Specific T Cell Responses

**Figure 4:**
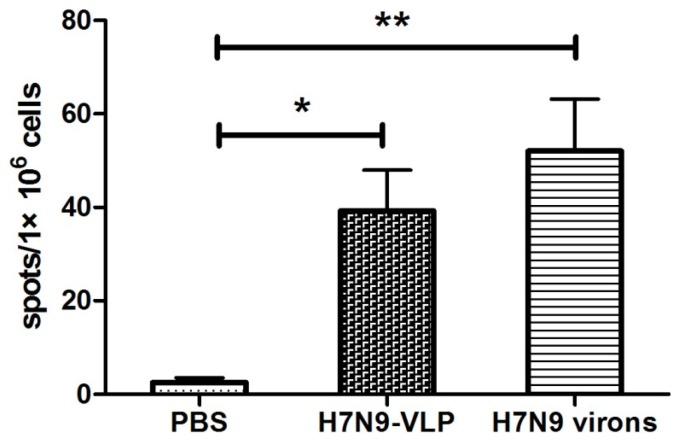
H7N9 specific T cell responses evaluated by IFN-γ ELISPOT assay. Mice were immunized with H7N9 VLPs, virions or PBS (n = 8 mice/group) for three times at two-week intervals. Spleen cells were collected from individual mouse and stimulated *in vitro* with H7N9 virions. ELISPOT assay was performed to test IFN-γ PRODUCTION. The mean number of spot forming cells/10^6^ splenocytes was shown with a S.D. bar. * indicates *p* < 0.05; ** indicates *p* < 0.01.

To examine specific T cell memory induced by H7N9 VLPs, an ELISPOT assay was employed to test virus specific cytotoxic T cell responses. At week 6 after the initial immunization, mice were sacrificed and spleen cells were collected. Each group of immunized spleen cells were *in vitro* stimulated with avian influenza H7N9 viruses A/Wuxi/1/2013 (H7N9) (GenBank: KF034914.1) for IFN-γ production. As shown in Figure 4, higher numbers of IFN-γ secreting splenocytes specific to the H7N9 virus were observed in the H7N9 VLPs immunized group as compared to control mice, and the extent was similar to the group of H7N9 virions immunized mice. These results demonstrated that vaccination with H7N9 VLPs could elicit specific cellular immune responses.

**Figure 5:**
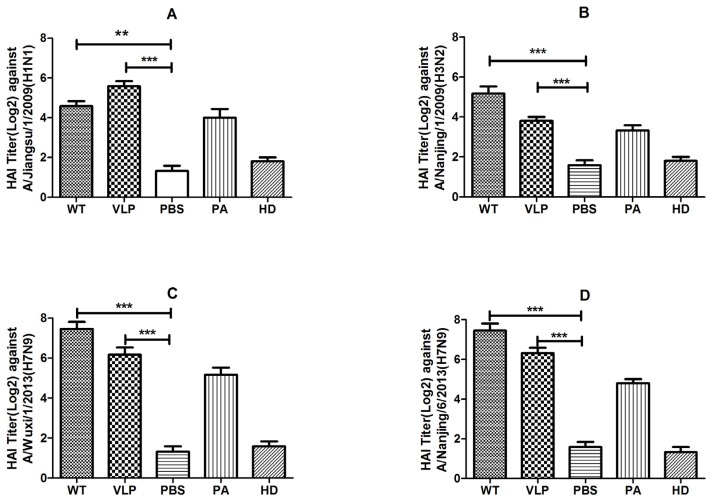
The hemagglutination inhibition antibody response to a panel of influenza virus. Immune sera were collected from sacrificed mice in each group (n = 8). Sera was serial diluted and 4 HA units of virus were added in v-bottom plates. After incubation, turkey erythrocytes were added. The HAI antibody titer was determined by the reciprocal dilution of the last row of non-agglutinated TRBC. HAI antibody titers against H7 subtype virus were measured by A/Wuxi/1/2013(H7N9) (**C**), and A/Nanjing/6/2013(H7N9) (**D**). Cross-immune reactions of H7 subtype VLP elicited antibodies were investigated by the HAI antibody titers to H1 subtype virus (**A**) and H3 subtype virus (**B**). HAI titers from each group were made numerical logarithmic transformation with a S.D. bar. ** indicates *p* < 0.01; *** indicates *p* < 0.001. WT, VLP and PBS: H7N9 virions, H7N9 VLPs, and PBS vaccinated mice serum. PA: Serum from a H7N9 infected patient. HD: Serum from a healthy donor.

### 2.4. VLPs Induced Hemagglutination–Inhibition and Neutralization Activity

**Figure 6:**
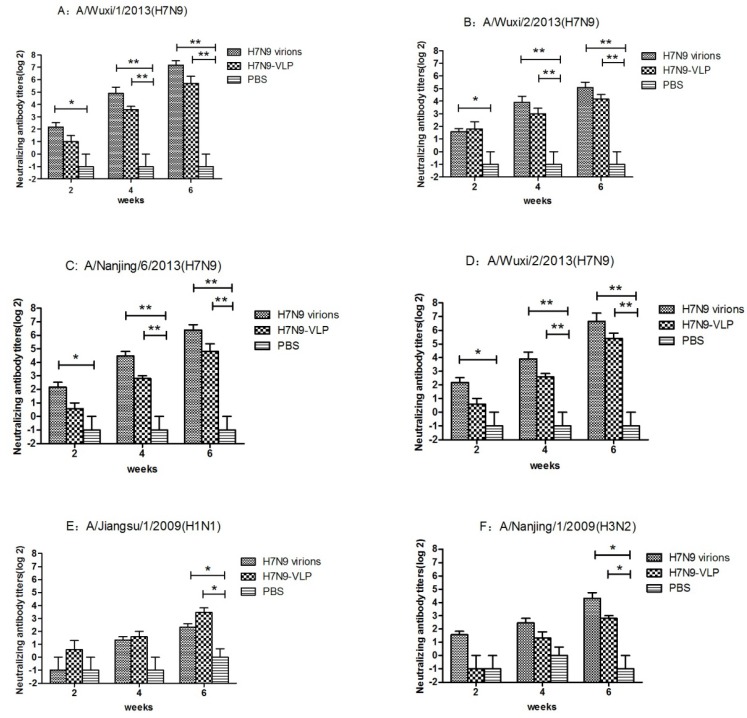
Serum neutralizing antibody titer of vaccinated mice. BALB/C mice were immunized with H7N9 VLPs, virions and PBS (n = 8 mice/group) for three times at two weeks interval. The mice were sacrificed at day 42 and the serum neutralizing antibodies specific to A/Wuxi/1/2013(H7N9) (**A**); A/Wuxi/2/2013(H7N9) (**B**); A/Nanjing/6/2013(H7N9) (**C**); A/Suzhou/3/2013(H7N9) (**D**); A/Jiangsu/1/2009 (H1N1) (**E**) and A/Nanjing/1/2009 (H3N2) (**F**) viruses were assessed by microneutralization assay. The cytopathic effect (CPE) was observed to determine neutralization titers at day 7. Data from each group were expressed as geometric mean titer (GMT) with a S.D. bar. * indicates *p* < 0.05; ** indicates *p* < 0.01. H7N9 virions, VLP and PBS: H7N9 virions, H7N9 VLPs, and PBS vaccinated mice serum.

The HAI antibody titers induced by vaccination with 40 μg of VLPs was up to 1:64 against the A/Wuxi/1/2013(H7N9). In contrast, mice vaccinated with the same dose of WIV developed HAI titers that were approximately twice as high as those with the VLPs (Figure 5C). No difference was found in the HAI antibody titer between A/Wuxi/1/2013(H7N9) and A/Nanjing/6/2013(H7N9) (Figure 5D). The cross-reactivity was observed by detecting the HAI antibody titer in the heterotype influenza virus. On A/Jiangsu/1/2009(H1N1) virus, the average HAI titer of VLPs group was about 1:50, which was twice as much as WIV’s (Figure 5A). While, the HAI titer of WIV against A/Nanjing/1/2009(H3N2) virus was roughly twice as high as VLPs (Figure 5B). Serum from an H7N9 infected patient (PA) and serum from a healthy donor (HD) were served as positive and negative control. Titers of PA and HD could be seen in each lane of Figure 5. Four strains of avian influenza H7N9 virus (A/Wuxi/1/2013(H7N9)), A/Wuxi/2/2013(H7N9), A/Nanjing/6/2013(H7N9) and A/Suzhou/3/2013(H7N9) and two strains of seasonal influenza A/Jiangsu/1/2009(H1N1) and A/Nanjing/1/2009(H3N2) were used to determine the neutralization ability of serum. As shown in Figure 6, both WIV and VLPs immunized mouse serum have a neutralization ability against four strains of avian influenza viruses (Figure 6A–D). In contrast, antiserum from VLP-immunized mice had lower neutralization titers (about 1:2), which increased by about 4-fold in week 4. By week 6, sera from both the VLP- and WIV-immunized mice showed high neutralization ability (1:32 or better) against H7N9 viruses. Cross-neutralizing activity was found in both WIV and VLPs immunized mouse serum against seasonal influenza A/Jiangsu/1/2009(H1N1) and A/Nanjing/1/2009(H3N2) (Figure 6E,F).

## 3. Discussion

Novel avian influenza A (H7N9) has caused an outbreak of severe human illness in China since March 2013. As a new reassortant virus, humans have little or no immunity to it [27], and there is no vaccine available for the H7 subtype. This has precipitated a global effort to rapidly develop and test H7N9 vaccine candidate.

In this research, we produce VLPs in 293T cells by co-transfection of three recombinant plasmids (HA-pcDNA5.0, NA-pcDNA5.0, and M1-pcDNA5.0). VLPs composed of HA, NA, and M1 of A/Wuxi/1/2013(H7N9) virus, can be generated in 293T cells. The self-assembly of VLP are dependent on the presence of M1 protein, which was previously shown to play the critical role in virus assembly and budding [35]. The size of 80–120 nm, and morphology, with spikes on the surface, of VLPs, confirm the self-assembly of VLP from another perspective. Afterwards, we have investigated detailed immune responses induced by influenza VLPs, including antibody isotypes, cross HAI antibody, and neutralizing activity, which have not been investigated previously. 

In our current study, full length, unmodified HA, NA, and M1 genes were derived from wild-type H7N9 virus to assemble VLPs. In previous studies, synthesized HA1 of H7N9 and M1 gene from either H5N1 or H3N2 were used to generate VLPs [18]. In contrast with the baculovirus-insect cell expression system, which was used in many influenza VLPs productions, mammalian cells were used to produce VLPs in this study. As produced in insect cells, recombinant HA cannot cleave into subunits of HA1 and HA2, which remain as the full-length of un-cleaved precursor (HA0). In mammalian cells, HA0 could be cut efficiently and assembled to natural conformation [28]. That makes our VLPs more similar to the WIV in antigenicity and morphology. The morphology of VLPs with spikes on their surfaces, characteristic of influenza virus HA proteins on virions were observed by electron microscopy.

In this manuscript, we found that the antibody titer increased significantly two weeks after vaccination. The dominant serum IgG isotype subclasses elicited by VLPs were IgG1, IgG2a as well as Kappa light chain. IgG2a was associated with Th1 immune response, which was demonstrated by testing specific cytotoxic T cell responses in the ELISPOT assay. In influenza A virus, the HA subtypes are classified into group 1(H1, H 2, H5, H6, H8, H9, H11, H12, H13, and H16) and group2 (H3, H4, H7, H10, H14, and H15) based on their sequence and antigenicity features [29]. Previous reports had shown that H7 subtype VLPs could elicit remarkable cross-reactivity to HA in both group 1 and group 2 [19]. This notion was confirmed by our study. By HAI assay, we observed H7N9 VLP could induce HAI antibody to both group 1 and group 2 viruses, where the HAI antibody titers were more than 1:16. Furthermore, microneutralization assay was utilized to determine the neutralization ability of serum *in vitro* in this paper*.* The cross-neutralizing antibodies to group 1 influenza virus and the ability to neutralize homotype and heterotype influenza viruses in group 2 were also detected in VLPs immunized mice. We found that VLPs could induce markedly neutralizing antibody titer against four strains of H7N9 viruses, which were isolated in Jiangsu province in 2013. A cross-neutralizing ability was also observed against H1N1 and H3N2 viruses.

VLPs are essentially “hollow-core” virus particles formed by self-assembly of HA, NA, and M1. It is generally accepted that VLPs that retain the structure and antigenicity of the infectious virions may have a better immunogenicity and be more immune protective than recombinant HA. By immunization, three structural proteins may have a synergistic effect on the immune response. VLPs could also induce a broader immune response than a corresponding whole-virion vaccine and have a more dominant Th1 response than a recombinant HA vaccine in mice and ferrets [30]. Research also proved that both HA and NA can stimulate protective immune responses including both humoral and cellular immunity in animals and humans [31]. VLPs also have the potential for activating both the endogenous and exogenous antigen pathways leading to the presentation of viral peptides by MHC class I and class II molecules. Particles, unlike single proteins, have the ability to bind and enter cells using appropriate surface receptors. VLPs can be processed and presented on MHC class I molecules, therefore promoting presentation to T-cells by professional antigen presenting cells [32]. In this paper, H7N9 VLPs could efficiently induce cellular and humoral immunoresponse specific to H7N9 virus.

In contrast to WIV, VLPs have no genomic component and are replication incompetent, and either antigenic drift or shift will not be observed in immunization of VLPs. Viral NS1 protein is known to counteract virus recognition and IFN-γ production, which will inhibit the innate immunity of host on the early defense against viral infections [33]. While VLPs composed of only three structural proteins without NS and other components can also activate innate immune via pathogen recognition receptors, perhaps because they retain the structural components of the virus [34]. Moreover, live-attenuated or formalin-inactivated vaccines are time consuming and each year can lead to delays in production resulting in late delivery of vaccine after the beginning of the influenza season [30].

Taken together, we successfully acquired H7N9 influenza virus VLPs, which consisted of major antigenic proteins of the virus and exhibited similar morphological features as natural virus particles.

## 4. Materials and Methods

### 4.1. Cells and Virus

293T cells and Madin-Daby canine kidney (MDCK) cells were both cultured in Dulbecco’s Modified Eagle Medium (DMEM; Gibco, Grand Island, NY, USA) supplemented with 10% fetal bovine serum (FBS), penicillin (100 U/mL) and streptomycin(100 μg/mL) at 37 °C with 5% CO_2_. 

Avian influenza H7N9 virusesA/Wuxi/1/2013 (H7N9) (GenBank: KF034914.1), A/Wuxi/2/2013(H7N9) (GenBank: KF034919.1), A/Nanjing/6/2013(H7N9) (GenBank: KF007119.1), A/Suzhou/3/2013(H7N9) (GenBank: KF007111.1), and seasonal influenza viruses A/Jiangsu/1/2009(H1N1), A/Nanjing/1/2009(H3N2) were isolated and kept by our laboratory. Viruses were propagated in the allantoic cavity of 10-day-old embroyonated chicken eggs. Five days after inoculation allantoic, fluids containing viruses were harvested, aliquoted and frozen at −80 °C until later use. All operations with live virus were carried out in a biosafety level-3 facility.

### 4.2. Construction of H7N9 VLP Expression Plasmids

The QIAamp Viral RNA kit (Qiagen, Santa Clarita, CA, USA) was used to extract genomic RNA of H7N9 virus from allantoic fluid of infected chicken eggs. The extracted RNA was subjected to reverse transcription PCR (RT-PCR) using Transcriptor High Fidelity cDNA Synthesis Kit (Roche, Penzberg, Germany) to generate cDNA. The HA gene was amplified with primers containing Kpn I and Xho I enzyme sites: F-HA-Kpn I, 5'-GGGGTACCCACCATGAACACTCAAATCCTG-3' and R-HA-Xho I, 5'-CCGCTCGAGTTATATACAAATAGTGCACC-3'. The NA gene was amplified using the primers: F-NA-Nhe I, 5'-CTAGCTAGCCACCATGAATCCAAATCAGAAG-3' and R-NA- Kpn I, 5'-GGGGTACCTTAGAGGAAGTACTCTATTTTAG-3'. The following primers were used for M1: F-M1-Nhe I, 5'-CTAGCTAGCCACCATGAGTCTTCTAACCGAG-3', and R-M1- Kpn I, 5'-GGGGTACCTCACTTGAACCGCTGCAGTTG-3'. The PCR segments were digested with specific enzymes and ligated with linearized pcDNA5/FRT vector. The nucleotide sequences of the HA-pcDNA5, NA-pcDNA5, and M1-pcDNA5 genes were confirmed by sequencing.

### 4.3. Transient Transfection of 293T Cells with H7N9 VLP Expression Plasmids

293T cells were seeded in 6-well plates with a density of 3 × 10^5^ per well one day before transfection. Recombinant plasmids containing HA-pcDNA5, NA- pcDNA5, and M1- pcDNA5 were co-transfected to cells by X-tremeGENE HP DNA Transfection Reagent (Roche) following manufacturer’s instruction. 48 h post-transfection, cells and supernatants were harvested for future use. 10 cm cell culture dishes were used for large-scale production of VLPs.

### 4.4. Western Blot Analysis

Culture supernatants were harvested without concentration, then electrophoresed in NuPAGE 4%–12% Bis-Tris gradient gel and transferred to nitrocellulose membrane (GE Healthcare, Piscataway, NJ, USA). Membranes were blocked by 10% skimmed milk for 1 h at room temperature (RT), and incubated with diluted H7N9 virus-specific mouse or human sera for another hour at RT. After intensive washing, the membranes were incubated with HRP- conjugated second antibodies, respectively. Protein bands were visualized by ECL plus Western blotting detection system kit (GE Healthcare). Radiographs were exposed, developed, and fixed according to the manufacturer’s instructions.

### 4.5. Electron Microscopy

Seventy-two hours post-transfection, 293T cells were fixed with 0.25% glutaraldehyde and 1% osmium tetraoxide, dehydrated with ethanol, and then embedded in epon resin. Thin sections were stained with lead citrate and uranyl acetate and observed byelectron microscope. 

### 4.6. Purification of H7N9-Derived VLPs

The culture supernatants containing extracellular VLPs were harvested and clarified by centrifugation at 10,000 *g* for 30 min followed by concentration using ultra-filtration system Startocon slice 200 (Sartorius stedim biotech, Aubagne, France). VLPs were pelleted for 4 h at 100,000 *g* at 4 °C using SW41 rotor (Beckman Coulter Inc., Fullerton, CA, USA). The pellets were re-suspended in 5 mL of PBS (pH 7.2), and loaded onto a 20%–60% (*w/v*) discontinuous sucrose step density gradient, and sedimented by ultracentrifugation for 6 h at 100,000 *g*. About 1 mL fractions were collected and pelleted by a second ultracentrifugation and then re-suspended in PBS. Total protein concentrations of VLPs were determined by Pierce BCA Protein Assay Kit.

### 4.7. Mice Immunization

Six-week-old female BALB/c mice were purchased from Model Animal Research Center of Nanjing University, China. Mice were randomly divided into three groups (8 mice per group). Mice were immunized intramuscularly with 40 μg (total protein) of VLPs or purified H7N9 whole influenza virions (WIV) (A/Wuxi/1/2013(H7N9)) in Freund’s complete adjuvant (Sigma) for priming and in Freund’s incomplete adjuvant for two times of boosting at an interval of 2 weeks. Negative control mice were immunized with PBS in the same adjuvant. On days 0, 14, and 28, blood samples were collected through the tails in for antibody detection. Two weeks after the last immunization, mice were sacrificed to collect serum for the neutralizing antibodies assay and hemagglutination inhibition (HAI) test. All experimental procedures were conducted in conformity with the National Institutes of Health Guide for Care and Use of Laboratory Animals (Publication No. 85-23, revised 1985). The protocol was approved by the animal care committee of JSCDC. Mice were sacrificed by cervical dislocation, and all efforts were made to minimize suffering.

### 4.8. ELISA to Measure Antibody Titer and Antibody Isotypes in Serum

Mice sera titer against VLPs was measured by an indirect ELISA. Firstly, H7N9 virus (A/Wuxi/1/2013) was inactivated by 0.05% beta-propiolactone treatment [36] and coated on 96-well plates (1:2000 diluted by carbonate-bicarbonate buffer, pH 9.6) at 4 °C overnight. After blocking, two-fold serial diluted serum samples (starting from 1:1000) were added and incubated at 37 °C for 1h. After that, goat anti-human antibody-HRP conjugate was added (1:2000, Sigma Aldrich, St. Louis, MO, USA). Then tetramethylbenzidine (TMB) substrate (Thermo Fisher, Waltham, MA, USA) was used for detection. The absorbance was read at 450 nm. The value which exceeds the mean + 2 standard deviation (S.D.) of negative control was considered positive.

For identification of the heavy and light chain isotypes of VLPs specific antibodies, the Clonotyping system-HRP kit (Souther Biotech, Birmingham, AL, USA, cat: 5300-05) was used. Influenza virus specific IgG1, IgG2a, IgG2b, IgG3, IgM, kappa chain and lambda chain antibodies were determined by ELISA as described previously [37].

### 4.9. Hemagglutination Inhibition (HAI) Titer and Neutralization Assay

The HAI and neutralization assay of mice VLP-specific sera were performed according to “Serological detection of avian influenza A (H7N9) virus infections by turkey haemagglutination-inhibition assay” [38] and “Serological detection of avian influenza A (H7N9) infections by microneutralization assay” [39] which were provided by WHO.

To evaluate the ability to prevent virus-induced agglutination of turkey RBCs, HAI antibody titers of vaccinated mice were tested by using two strains of H7 subtype virus (A/Wuxi/1/2013(H7N9) and A/Nanjing/6/2013(H7N9)). Cross-immune reactions of H7N9 VLPs elicited antibodies were investigated by determining HAI antibody titers to one strain of H1 subtype virus (A/Jiangsu/1/2009(H1N1)), and one strain of H3 subtype virus (A/Nanjing/1/2009(H3N2)).

Four strains of avian influenza H7N9 virus (A/Wuxi/1/2013(H7N9), A/Wuxi/2/2013(H7N9), A/Nanjing/6/2013(H7N9) and A/Suzhou/3/2013(H7N9)) were used to determine the neutralization ability of serum. For microneutralization assay, every dilution of each serum sample was performed in quadruplicate. The neutralizing antibody titer was expressed as the maximum serum dilution at which CPE was not observed in all four wells.

### 4.10. Enzyme Linked Immunospot (ELISPOT) Assay

The ELISPOT 96-well plates (BD, San Jose, CA, USA) were coated with 100 μL of anti-mouse IFN-γ (5 µg/mL in coating buffer) at 4 °C overnight. The following day, plates were washed and blocked with blocking buffer for 2 h at 37 °C. Then, 100 μL freshly isolated splenocytes (5 × 10^5^ cells) from the immunized mice were added to each well and stimulated with avian influenza H7N9 viruses A/Wuxi/1/2013 (H7N9) (GenBank: KF034914.1) at 37 °C for 40 h with 5% CO_2_. After that, cells were washed, biotinylated anti-mouse IFN-γ was added to each well and incubated for 2 h at room temperature. Then plates were washed and incubated for 1 h at room temperature with streptavidin-HRP. Finally, AEC substrate solution (BD) was added and spots were counted by ImmunoSpot Analyzer (Cellular Technology Ltd., Cleveland, OH, USA).

### 4.11. Statistical Analysis

Statistical significance of the antibody isotype and the serum neutralizing antibody titer datum were assessed using the one-way ANOVA and Tukey HSD test. A *p*-value < 0.05 was considered significant. Statistical analyses were done using GraphPad Prism 5.0 software.

## 5. Ethics Statement

All experimental procedures were conducted in conformity with the National Institutes of Health Guide for Care and Use of Laboratory Animals (Publication No. 85-23, revised 1985). The protocol was approved by the animal care committee of Jiangsu Provincial Center for Disease Prevention and Control, Nanjing, China. Mice were sacrificed by cervical dislocate, and all efforts were made to minimize suffering.
